# Opposition-based chaotic evolution for optimization

**DOI:** 10.1038/s41598-025-09207-4

**Published:** 2025-07-02

**Authors:** Tianshui Li, Yan Pei

**Affiliations:** 1 Graduate School of Computer Science and Engineering, University of Aizu, Aizu-Wakamatsu, 965-8580 Japan; 2 Computer Science Division, University of Aizu, Aizu-Wakamatsu, 965-8580 Japan

**Keywords:** Evolutionary computation, Chaotic evolution, Opposition-based learning, Single-objective optimization, Multi-objective optimization, Conceptual hybrid rocket engine design, Computational science, Computer science

## Abstract

Evolutionary computation algorithms are widely used for solving complex optimization problems, but their practical performance is often hindered by high computational costs and premature convergence. To address this challenge, we propose an opposition-based chaotic evolution (OBCE) algorithm that integrates opposition-based learning (OBL) into the chaotic evolution framework. The OBL mechanism introduces mirrored solutions to increase population diversity and improve global search ability with minimal overhead. The proposed algorithm is evaluated on a series of single-objective and multi-objective numerical optimization problems, including standard benchmark functions and a real-world hybrid rocket engine design task. Compared to conventional chaotic evolution and other baseline algorithms, OBCE consistently shows faster convergence and better solution quality across various problem types and dimensions. In multi-objective settings, OBCE enhances the diversity of Pareto solutions, offering broader decision-making options. In the rocket engine design task, the algorithm finds more competitive design parameters under realistic constraints. These results demonstrate that integrating OBL into chaotic evolution can effectively mitigate premature convergence and improve optimization performance in both theoretical and applied scenarios. The findings support the broader applicability of OBCE in real-world engineering design problems where solution quality and efficiency are critical.

## Introduction

In real life, almost every problem in science and engineering can be converted into a single-objective or multi-objective optimization problem in some appropriate ways. The goal of a single-objective problem is to select the optimal solution based on a specific criterion or metric from all possible options of a problem. Most of the real-world problems are more likely to be multi-objective optimization problems with highly complex and non-linear characteristics. Unlike the single-objective optimization problem, which pursues only one goal, the multi-objective optimization problem aims at finding many Pareto optimal solutions that are generated from a trade-off among multiple conflicting objectives. There is no single solution that simultaneously optimizes each objective in the usual multi-objective optimization problem.

Due to the creativity and stochasticity characteristics of EC algorithms, they are considered effective methods for solving both single-objective and multi-objective optimization problems. EC algorithms mimic natural principles of biological evolution and swarm intelligence to achieve global optimization, such as genetic algorithm^1^, differential evolution^2^, particle swarm optimization^3^, ant colony optimization^4^, fireworks algorithm^5^, artificial bee colony^6^, and grey wolf optimizer^7^. In recent decades, EC algorithms have demonstrated good performance on many optimization problems^8–11^, attracting numerous researchers to work on improving optimization performance within the EC community^12–15^. In EC algorithms, the necessary condition for algorithm convergence is to maintain the optimal solution set of the previous generation and utilize it during the evolution process of the new generation. This approach allows the optimal solution set of the evolutionary population to continue converging to the local and/or global optimal gradually, ultimately resulting in a satisfying and promising solution set.

To thoroughly investigate EC algorithms, it requires both artful search and selection. A robust search capability and an intelligent selection strategy play pivotal roles in optimization performance. Chaotic evolution is a novel evolutionary computation algorithm that integrates the iteration of EC with the ergodicity of chaos^16^. A series of comparisons and investigations have been conducted to demonstrate that chaotic evolution exhibits strong optimization performance, particularly for higher-dimensional problems^16,17^.

Opposition-based learning (OBL)^18^ is a lightweight scheme in machine intelligence. Since its fundamental concept was first proposed by Tizhoosh, it has garnered significant research attention. The OBL mechanism employs a straightforward approach to effectively search potential regions, thereby enhancing the diversity of the search population and global search capability. In recent years, numerous opposition-based EC algorithms have been proposed to enhance their search capabilities and optimization performance, including opposition-based particle swarm optimization^19,20^, opposition-based differential evolution^21^, opposition-based artificial bee colony^22^, and opposition-based chaotic evolution^23^.

This study extends previous work^23^ and further evaluating a new chaotic evolution algorithm, opposition learning-based chaotic evolution algorithm (OBCE), which integrates the OBL mechanism into the conventional chaotic evolution algorithm. In the proposed algorithm, the OBL mechanism is incorporated into the conventional chaotic evolution algorithm to generate opposite vectors during its search process. Both chaotic vectors and opposite vectors are considered when determining the offspring individuals for the next generation. Based on previous work^23^, the algorithm is further applied to multi-objective optimization problems and practical application in this study. Additionally, supplementary experiments are conducted to track and rank the opposition-based population, aiming to more specifically assess the effectiveness of the OBL mechanism within the proposed framework. The effectiveness of the OBL mechanism in the proposed OBCE algorithm is assessed and analyzed from two perspectives: single-objective optimization problems and multi-objective optimization problems. Based on the experimental results, it is concluded that integrating the OBL mechanism into the conventional chaotic evolution algorithm increases the likelihood of alleviating premature convergence. For single-objective optimization problems, the proposed OBCE algorithm accelerates convergence speed and demonstrates superior optimization performance. Regarding multi-objective optimization problems, the proposed OBCE algorithm enhances the diversity of Pareto solutions, thereby offering decision-makers a wider range of choices.

The subsequent sections of this paper are structured as follows: “Chaotic evolution” and “Opposition-based learning” provide an overview of the chaotic evolution algorithm and the OBL mechanism, respectively, while “Opposition learning-based chaotic evolution” elaborates on the details of the proposed algorithm (OBCE). Following this, “OBCE for single-objective optimization”, “OBCE for multi-objective optimization” and “OBCE for application in design of hybrid rocket engine” present experimental evaluations and discussions to address the effectiveness of the OBL mechanism on the chaotic evolution algorithm’s optimization performance across single-objective optimization problems, multi-objective problems, and real-world applications. Furthermore, “Discussion on mechanism of opposition-based learning” offers a more in-depth analysis of the role of the OBL mechanism within the proposed framework. Finally, “Conclusion and future work” concludes the study and outlines potential directions for future research.

## Chaotic evolution

Chaotic evolution is a novel EC algorithm that incorporates a mathematical mechanism, known as the chaotic ergodic property, into the evolutionary optimization process. The principle of chaotic evolution is to simulate chaotic ergodic motion for implementing search in the optimization^16^. This algorithm utilizes the ergodic property of chaos to conduct both exploration and exploitation functions within an evolutionary framework, thereby ensuring robust optimization capabilities. In chaotic evolution, the mutant operation parameters chaotic parameter and direction factor according to a direction factor rate are generated before generating mutant vector operation. Subsequently, the mutant vector is produced from the target vector using Eq. (1). Following this, the chaotic vector is obtained by combining the target vector and the mutant vector through a crossover process governed by the crossover rate, as described in Eq. (2).

1 $$\begin{aligned} mutant_i= & target_i * (1 + D_i * CP_i) \end{aligned}$$

2 $$\begin{aligned} chaotic_i= & Crossover(target_i; mutant_i) \end{aligned}$$

One crucial difference between single-objective chaotic evolution and multi-objective chaotic evolution lies in the selection strategy. As previously mentioned, the objective of a single-objective problem is to select the optimal solution based on a specific criterion or metric from all possible options. Consequently, the selection strategy in the single-objective chaotic evolutionary algorithm primarily emphasizes fitness value. Conversely, in a typical multi-objective optimization problem, there is no single solution that optimizes each objective simultaneously. In this scenario, non-dominant sorting and tournament selection using crowding distance are introduced to the selection strategy of MOCE. This is aimed at addressing the Pareto dominance issue and maintaining solution diversity.

The primary steps of MOCE implementation are as follows:

1. Initialization: Randomly initialize a population $\{{\textbf{x}}_i^0\}_{i=1}^{PS}$, where *PS* is the population size.
2. Parameter Generation: For each individual, generate a direction factor $D_{i,j}^G \in \{-1, +1\}$ and a chaotic parameter $CP_{i,j}^G \in (0,1)$ using a chaotic map (e.g., logistic map):
  3 $$\begin{aligned} CP_{i,j}^{G+1} = \mu \cdot CP_{i,j}^{G} \cdot (1 - CP_{i,j}^{G}), \quad \mu = 4 \end{aligned}$$
3. Mutation: For each individual ${\textbf{x}}_i^G$, generate a mutant vector ${\textbf{v}}_i^G$ as:
  4 $$\begin{aligned} v_{i,j}^G = x_{i,j}^G \cdot \left( 1 + D_{i,j}^G \cdot CP_{i,j}^G \right) \end{aligned}$$
4. Crossover: Create a chaotic vector ${\textbf{c}}_i^G$ by combining ${\textbf{x}}_i^G$ and ${\textbf{v}}_i^G$ through binomial crossover:
  5 $$\begin{aligned} c_{i,j}^G = {\left\{ \begin{array}{ll} v_{i,j}^G, & \text {if } \text {rand}(0,1) < Cr \text { or } j = j_{\text {rand}} \\ x_{i,j}^G, & \text {otherwise} \end{array}\right. } \end{aligned}$$
  where *Cr* is the crossover rate and $j_{\text {rand}}$ is a randomly chosen index to ensure at least one crossover occurs.
5. Evaluation: Evaluate the fitness vector ${\textbf{F}}({\textbf{c}}_i^G) = [f_1({\textbf{c}}_i^G), f_2({\textbf{c}}_i^G), \dots , f_M({\textbf{c}}_i^G)]$ for all individuals ${\textbf{c}}_i^G$ in the offspring population, where *M* is the number of objectives.
6. Selection: Combine parent and offspring populations, then select the next generation using the fast non-dominated sorting and crowding distance mechanism.
7. Termination: Repeat steps (2)–(6) until the maximum number of generations is reached.

In the aforementioned primary steps, the target vector corresponds to an individual within the current generation population. The mutant vector, on the other hand, is generated by simulating chaotic motion from a chaotic system. The ergodicity of chaotic system can support more diversity to enhance exploration and exploitation capabilities of evolutionary algorithms^17^. The distribution of the chaotic system plays a pivotal role in the optimization performance of the chaotic evolution algorithm. Therefore, selecting a suitable chaotic system is crucial for the chaotic evolution algorithm.

## Opposition-based learning

OBL^18^ represents a scheme designed to expedite optimization search using a simple mathematical mechanism. By applying this mechanism to both the population initialization process and the generation of new offspring, EC algorithms demonstrate enhanced optimization performance. The efficiency of integrating the OBL mechanism to algorithms such as particle swarm optimization algorithm^19,20^, differential evolution algorithm^21^, artificial bee colony algorithm^22^, and chaotic evolution algorithm^23^ has already been demonstrated. The mathematical definition of this mechanism is as follows. Suppose *x* is a real number within an interval [*a*, *b*] (*x* $\in$ [*a*, *b*]), the opposite number of *x*, denoted as *OP*(*x*), is defined by Eq (6).

6 $$\begin{aligned} OP(x) = a + b - x. \end{aligned}$$

In this context, consider a point *X* in an *n*-dimensional real number space, denoted as *X* = ($x_1$, $x_2$, ..., $x_n$) (where $x_i$ $\in$ $[a_i, b_i]$, for $i = 1, 2,\ldots , n$; and $a_i$, $b_i$ $\in$ *R*). The opposite point of *X*, *OP*(*X*), is defined by Eqs. (7) and (8) respectively.

7 $$\begin{aligned} OP(X)= & (OP(x_1), OP(x_2),\ldots , OP(x_n)). \end{aligned}$$

8 $$\begin{aligned} OP(x_i)= & a_i + b_i - x_i. \end{aligned}$$

In general, heuristic optimization algorithms, such as EC algorithms, typically initialize their populations randomly. When the initial random guess is close to the optimal solution, the computational time for algorithm convergence is significantly reduced. However, it’s evident that initial populations are often distant from the optimal solution, particularly in complex problems. In such cases, simultaneously checking the opposite population can increase the likelihood of reducing the distance between trial solutions and optimal solutions^18^.

## Opposition learning-based chaotic evolution

In research on single-objective evolutionary algorithms, accelerating convergence speed and improving optimization performance are crucial areas of study. A faster convergence speed allows algorithms to yield better solutions at the same computational cost. Similarly, in the realm of multi-objective evolutionary algorithms, the convergence and diversity of Pareto solutions stand out as significant research topics. In the proposed algorithm, OBCE, the OBL mechanism is integrated into the conventional chaotic evolution algorithm and leverage the logistic map as a chaotic system to enhance solution diversity and increase the likelihood of alleviating premature convergence.

The evolutionary algorithm can be understood as an algorithm based on the generate-and-test search framework. To enhance its search capability, more effort is required in two aspects: search step size and search direction. Within the chaotic evolution optimization framework, the chaotic system plays a crucial role in controlling the search step size. Therefore, it’s evident that different choices of chaotic systems will significantly affect the optimization performance of the chaotic evolution algorithm. Any non-linear system with chaotic output characteristics can be utilized as a chaotic system.

The logistic map is employed as the chaotic system to update the chaotic parameters in the proposed methods. Equation (9) presents its mathematical expression, where $\mu$ is a parameter that determines its system outputs. In this study, $\mu$ is set to 4 for both conventional chaotic evolution algorithms and the proposed OBCE algorithms. When $\mu$ is set to 4, the logistic map generates outputs that are primarily concentrated in the intervals (0, 0.1] and (0.9, 1], with frequencies of approximately $20.55\%$ and $20.52\%$, respectively^17^. When generating a mutant vector based on a target vector, smaller chaotic parameters within the range of (0, 0.1] assist in exploiting areas near the target vector, whereas larger chaotic parameters in the range of (0.9, 1] facilitate exploration of distant regions. This mechanism enables the chaotic evolution algorithm to more effectively locate local or global optima, thereby accelerating convergence while maintaining solution diversity through deeper search space coverage.

9 $$\begin{aligned} X_n = \mu * X_{n-1} * (1 - X_{n-1}). \end{aligned}$$

Furthermore, the OBL mechanism is integrated into the conventional chaotic evolution algorithm to promote innovation and enhancement in the search direction. During the search process, if the search range of a particular individual is insufficient to escape the vicinity dominated by its local optimum, it may engage in unnecessary and ineffective computations persistently until the final generation. To mitigate this limitation, the incorporation of the OBL mechanism increases the likelihood of alleviating premature convergence and enhancing solution diversity.

In the proposed OBCE algorithm, the population initialization process begins with the generation and evaluation of a random initial population. In each generation, chaotic vectors are obtained based on the target vectors, chaotic parameters, and crossover rate. Subsequently, the opposite vectors of the chaotic vectors are generated using the OBL mechanism. The lower ranges, $a_j$, and upper ranges, $b_j$, are defined according to the respective ranges of the optimized variables. The fitness of each individual in both the chaotic population and the opposition-based population is then evaluated. Satisfactory individuals are selected as the target population for the next generation and passed on to the next iteration until the stopping criterion is met. As previously mentioned, the pseudo-code of the proposed opposition learning-based multi-objective chaotic evolution (OBMOCE) algorithm is shown in Algorithm 1.

**Algorithm 1:**
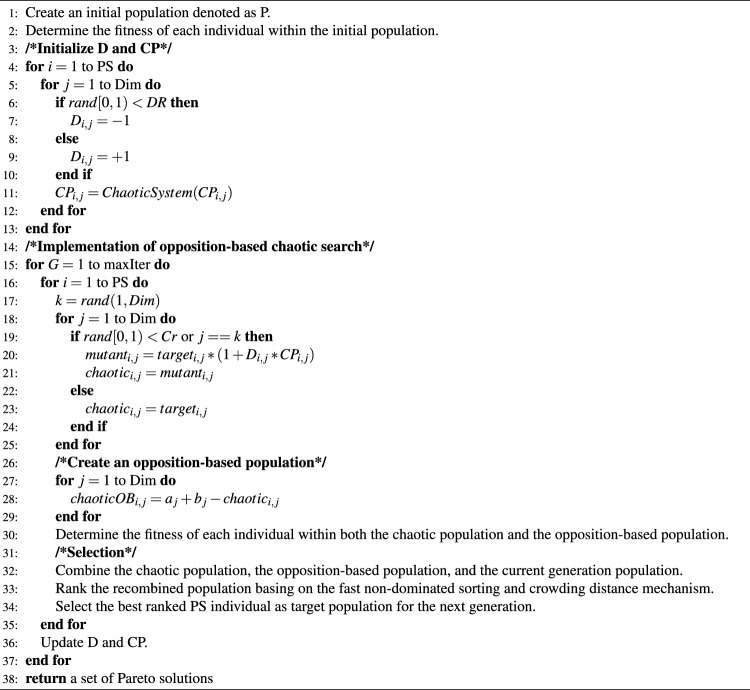
Opposition Learning-based Multi-objective Chaotic Evolution. PS: population size; Dim: dimension; D: direction factor; DR: direction factor rate; CP: chaotic parameter; G: generation; maxIter: maximum generation; i: index of individual; j: index of dimension; [$a_j$, $b_j$]: range of the $j^{th}$ variable.

## OBCE for single-objective optimization

### Experimental setting

To evaluate the effectiveness of the OBL mechanism in the proposed OBCE algorithm for the single-objective optimization problems, a comparative analysis is conducted among the conventional chaotic evolution algorithm and three proposed OBCE algorithms. These proposed OBCE algorithms incorporate opposite vectors into the chaotic evolution framework in different ways: the opposite vector of the target vector, the opposite vector of the chaotic vector, and both the opposite vectors of the target and chaotic vectors. The abbreviations for these comparison algorithms are presented in Table 1.

**Table 1:** The abbreviations of the algorithms used in evaluation.

Abbreviation	Meaning
CE	Ordinary chaotic evolution / pair comparison of a target vector and a chaotic vector using logistic map chaotic system
CEtOB	Triple comparison-based chaotic evolution among target, chaotic, target-OB using logistic map chaotic system
CEcOB	Triple comparison-based chaotic evolution among target, chaotic, chaotic-OB using logistic map chaotic system
CEtcOB	Quadruple comparison-based chaotic evolution among target, chaotic, target-OB, chaotic-OB using logistic map chaotic system

Target-OB and chaotic-OB refer to the opposition vectors for target vectors and chaotic vectors

The experiments were implemented under 2-dimensional, 10-dimensional, and 30-dimensional settings to evaluate the effectiveness of the OBL mechanism on the optimization performance of the chaotic evolution algorithm in increasingly complex single-objective optimization problems. 12 well-known benchmark functions from CEC 2005^24^ were employed to assess the optimization performance of the proposed methods. These functions exhibit diverse characteristics, including uni-modal, multi-modal, separable, non-separable, shifted, and rotated properties. This diversity enables a more comprehensive and effective evaluation of the contribution of the OBL mechanism to the performance of the chaotic evolution algorithm.

For each function, 30 independent runs were conducted, with 1000 generations per run. As previously described, these benchmark functions were used to compare the proposed methods with the conventional chaotic evolution algorithm under three different population size settings: PS = 10, PS = 50, and PS = 150 for Dim = 2, Dim = 10, and Dim = 30, respectively. The experimental parameters for these four compared methods are detailed in Table 2.

**Table 2:** Experimental parameters setting for single-objective optimization problems in this study.

Population size	10, 50, 150
Max. search generation	1000
Dimensions of benchmark function	2, 10, 30
Direction factor rate	0.5
Crossover rate	0.9
No. of trial runs	30

### Results and discussion

The computational cost associated with fitness value calculations clearly dominates the total run time of the algorithm. As a result, the number of fitness calculations serves as a critical performance index. Given that the number of fitness evaluations differs across generations between the proposed OBCE algorithms and the conventional chaotic evolution algorithm, a direct comparison based on generation count would be inappropriate. Therefore, in performance comparison and evaluation, the number of fitness evaluations is used as the baseline metric.

**Figure 1:**
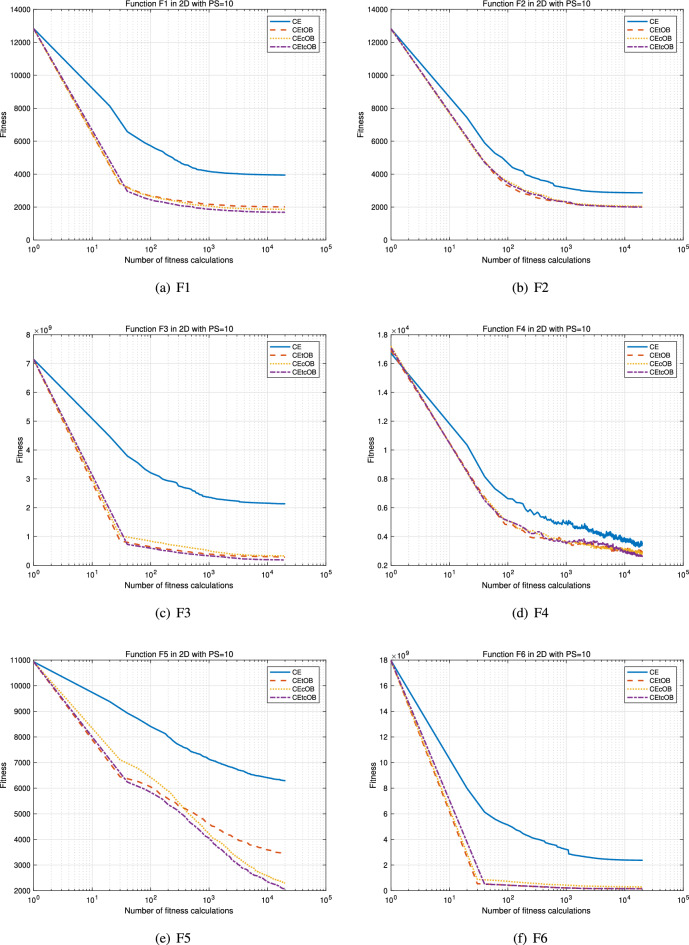
Average convergence curves for 30 running trials with Dim = 2, PS = 10 for F1–F6. The newly proposed algorithms have a better search capability to accelerate optimization.

**Figure 2:**
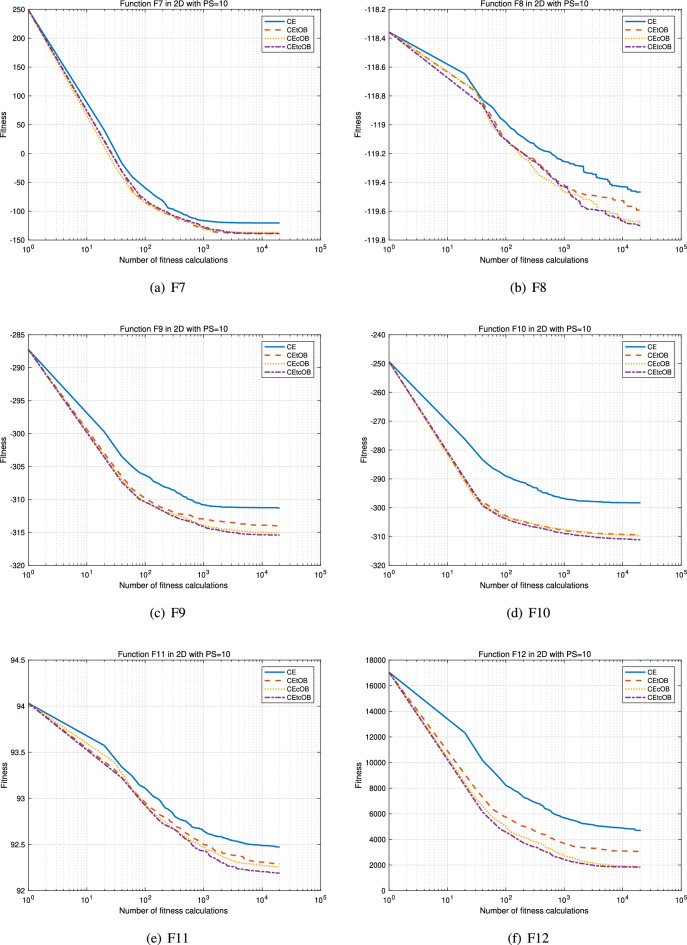
Average convergence curves for 30 running trials with Dim = 2, PS = 10 for F7–F12. The newly proposed algorithms have a better search capability to accelerate optimization.

Figures 1 and 2 present the convergence curves of F1–F12 for the 2-dimensional benchmark setting. It is evident that the proposed methods CEtOB, CEcOB, and CEtcOB exhibit superior optimization performance compared to the conventional method chaotic evolution when evaluated under the same numbers of fitness calculations, especially the proposed method CEtcOB and CEcOB. Additionally, the incorporation of the OBL mechanism enhances the search capability of the conventional chaotic evolution algorithm, thereby accelerating optimization.

Tables 3, 4, and 5 present the mean fitness values obtained from 30 independent trials, each evaluated using the same number of fitness evaluations, specifically 19,920 times. Due to the relatively small population sizes used, it is inherently challenging to reach the global optima in such settings of 2-dimensional, 10-dimensional, and 30-dimensional problems with only 10 individuals, 50 individuals, and 150 individuals, respectively. However, the average fitness values achieved by the proposed algorithms are generally much closer to the global optima compared to those obtained by the conventional chaotic evolution algorithm. To assess the statistical significance of the performance differences, the Wilcoxon signed-rank test is employed at a significance level of $\alpha < 0.05$. This non-parametric test is used to compare two related samples and determine whether a significant difference exists between them. As shown in Tables 3, 4, and 5, the proposed CEcOB and CEtcOB algorithms significantly outperform the conventional chaotic evolution algorithm across all 12 benchmark functions under the three different search conditions. Furthermore, when comparing the three proposed OBCE algorithms, both CEcOB and CEtcOB algorithms exhibit significantly better performance than the CEtOB algorithm across all 12 benchmarks in the 10-dimensional and 30-dimensional search conditions. A plausible explanation for this observation is that, in the CEtOB proposal method, when a target vector becomes trapped in a local optimum or converges slowly, it is likely to remain unchanged over many generations. As a result, its corresponding opposite vector also remains static, leading to redundant and ineffective computations. By contrast, the CEcOB and CEtcOB methods address this issue effectively. Leveraging the ergodic property of chaotic operations, even if the target vector remains unchanged, the chaotic vector and its corresponding opposite chaotic vector will continue to update, increasing the likelihood of escaping local optima and continuously exploring potential regions. Based on these results, the CEcOB algorithm is selected as the representative OBCE method for evaluating the effectiveness of the OBL mechanism in subsequent multi-objective optimization experiments.

**Table 3:** Mean and Wilcoxon signed-rank test results for 2-dimensional benchmark functions.

	F1	F2	F3	F4	F5	F6
CE	3.9422E+03	2.8674E+03	2.1365E+09	3.5392E+03	6.2918E+03	2.3730E+09
CEtOB	2.0123E+03†	2.0026E+03†	2.8518E+08†	3.0044E+03	3.4334E+03†	1.5966E+08†§
CEcOB	1.8628E+03†	2.0518E+03†	3.2722E+08†	2.8596E+03†	2.3009E+03†‡	2.9588E+08†
CEtcOB	1.6846E+03†‡§	1.99997E+03†	1.8321E+08†‡§	2.6475E+03†	2.0658E+03†‡§	1.4892E+08†§

	F7	F8	F9	F10	F11	F12
CE	− 1.2030E+02	− 1.1947E+02	− 3.1130E+02	− 2.9831E+02	9.2473E+01	4.6998E+03
CEtOB	− 1.3894E+02†	− 1.1959E+02	− 3.1399E+02†	− 3.0945E+02†	9.2289E+01†	3.0546E+03†
CEcOB	− 1.3683E+02†	− 1.1968E+02†	− 3.1506E+02†‡	− 3.0961E+02†	9.2259E+01†	1.8694E+03†‡
CEtcOB	− 1.3866E+02†§	− 1.1970E+02†	− 3.1540E+02†‡	− 3.1109E+02†‡§	9.2192E+01†‡§	1.8365E+03†‡

$\dag$, $\ddag$, $\S$, and $\pounds$ represent algorithms that are significantly better than CE, CEtOB, CEcOB, and CEtcOB, respectively, according to the Wilcoxon signed-rank test ($p < 0.05$)

**Table 4:** Mean and Wilcoxon signed-rank test results for 10-dimensional benchmark functions.

	F1	F2	F3	F4	F5	F6
CE	2.6436E+04	4.7124E+04	1.1368E+09	6.8245E+04	2.4055E+04	1.5173E+10
CEtOB	2.4793E+04†	3.5542E+04†	8.6313E+08†	6.4099E+04†	2.3062E+04†	1.3049E+10†
CEcOB	2.3891E+04†‡	3.3855E+04†‡	7.8591E+08†‡	6.0605E+04†‡	2.2285E+04†‡	1.2197E+10†‡
CEtcOB	2.3919E+04†‡	3.2912E+04†‡§	7.7484E+08†‡	6.1474E+04†‡	2.2266E+04†‡	1.2162E+10†‡

	F7	F8	F9	F10	F11	F12
CE	9.9467E+03	− 1.1895E+02	− 1.9201E+02	− 1.2663E+02	1.0468E+02	3.5797E+05
CEtOB	9.1479E+03†	− 1.1895E+02	− 1.9896E+02†	− 1.4187E+02†	1.0439E+02†	2.8764E+05†
CEcOB	8.7618E+03†‡	− 1.1901E+02†‡£	− 2.0175E+02†‡	− 1.4731E+02†‡	1.0433E+02†	2.8253E+05†
CEtcOB	8.8004E+03†‡	− 1.1900E+02†‡	− 2.0175E+02†‡	− 1.4736E+02†‡	1.0424E+02†‡	2.7857E+05†‡

$\dag$, $\ddag$, $\S$, and $\pounds$ have the same meanings as in Table 4

**Table 5:** Mean and Wilcoxon signed-rank test results for 30-dimensional benchmark functions.

	F1	F2	F3	F4	F5	F6
CE	8.9393E+04	5.6933E+05	2.8989E+09	8.5368E+05	5.6243E+04	5.3328E+10
CEtOB	8.8364E+04†	3.4034E+05†	2.7806E+09†	7.0087E+05†	5.2390E+04†	5.2994E+10
CEcOB	8.7017E+04†‡£	3.1345E+05†‡	2.6935E+09†‡	6.9194E+05†	5.0605E+04†‡	5.0022E+10†‡£
CEtcOB	8.7552E+04†‡	2.9668E+05†‡§	2.7048E+09†‡	6.4922E+05†‡§	5.0477E+04†‡	5.1193E+10†‡

	F7	F8	F9	F10	F11	F12
CE	4.4685E+03	− 1.1864E+02	1.8379E+02	5.7317E+02	1.4014E+02	2.3765E+06
CEtOB	4.3672E+03†	− 1.1864E+02	1.7566E+02†	5.5045E+02†	1.3971E+02†	2.3188E+06†
CEcOB	4.2723E+03†‡	− 1.1866E+02†‡	1.6745E+02†‡£	5.3448E+02†‡£	1.3956E+02†‡	2.2870E+06†‡
CEtcOB	4.2838E+03†‡	− 1.1866E+02†‡	1.6872E+02†‡	5.3773E+02†‡	1.3933E+02†‡§	2.2958E+06†‡

$\dag$, $\ddag$, $\S$, and $\pounds$ have the same meanings as in Table 4

## OBCE for multi-objective optimization

### Experimental setting

To evaluate the effectiveness of the OBL mechanism in the proposed OBMOCE algorithm, a comparative analysis is conducted among the proposed algorithm, the conventional MOCE algorithm^25^, and NSGA-II^26^ in this experiment. The abbreviations of the investigated algorithms are summarized in Table 6. The multi-objective test problems used in this experiment include 5 ZDT series test functions (ZDT1, ZDT2, ZDT3, ZDT4, ZDT6)^27^ and 5 DTLZ series test functions (DTLZ1, DTLZ2, DTLZ3, DTLZ4, DTLZ7)^28^. These ten benchmark problems exhibit diverse characteristics such as convexity, non-convexity, and disconnected Pareto fronts, providing a comprehensive assessment of algorithmic performance across various problem structures.

**Table 6:** The abbreviations of the algorithms used in evaluation.

Abbreviation	Meaning
NSGA-II	Non-dominated sorting genetic algorithm II
MOCE	Multi-objective chaotic evolution algorithm using logistic map chaotic system
OBMOCE	Opposition learning-based multi-objective chaotic evolution algorithm using logistic map chaotic system

For each of the benchmark functions, 30 independent trials were performed. The population size was set to 100, the crossover rate to 0.9, and the direction factor rate to 0.5. The ZDT-series test functions (ZDT1, ZDT2, ZDT3, ZDT4, ZDT6) are two-objective problems with dimensionalities of 30, 30, 30, 10, and 10, respectively. The DTLZ-series test functions (DTLZ1, DTLZ2, DTLZ3, DTLZ4, DTLZ7) used in this study are three-objective problems, each with a dimensionality of 30. In each generation, the proposed algorithm OBMOCE calculates the fitness of both the chaotic population and the opposition-based population. As a result, the computational cost of OBMOCE is twice that of the other two comparison algorithms. To ensure a fair comparison of optimization performance, the maximum number of generations was set to 1000 for both NSGA-II and MOCE, and 500 for OBMOCE. The experimental parameters for these three algorithms are detailed in Table 7.

**Table 7:** Experimental parameters setting for multi-objective optimization problems in this study.

Population size	100
Max. search generation	500, 1000
Dimensions of benchmark function	10, 30
Direction factor rate	0.5
Crossover rate	0.9
Num. of trial runs	30

### Results and discussion

**Figure 3:**
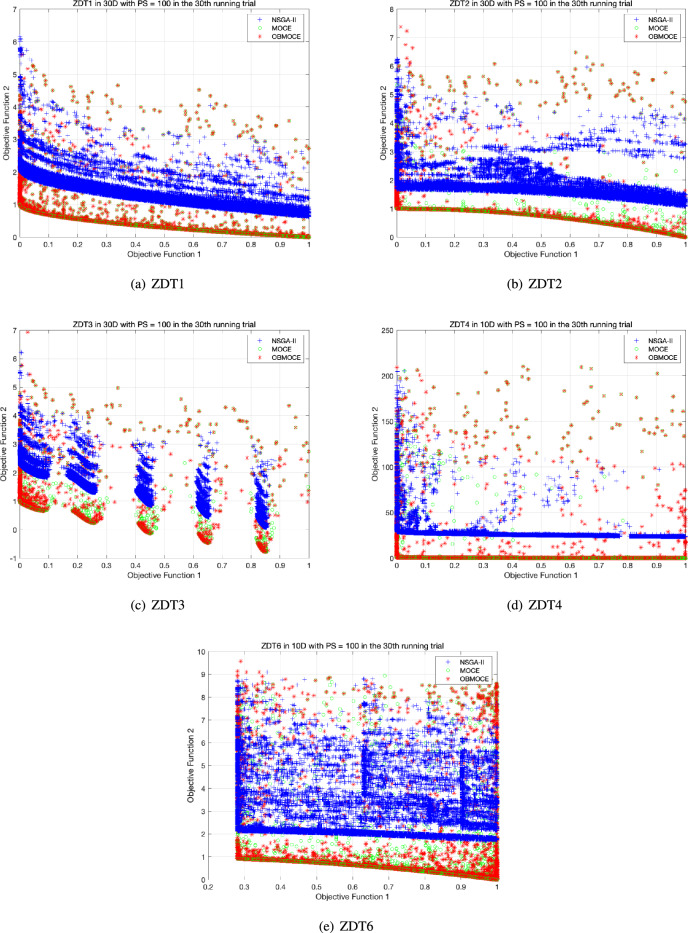
The distribution of solutions derived by three investigated algorithms across all generations from the 30th running trial for ZDT series test functions.

**Figure 4:**
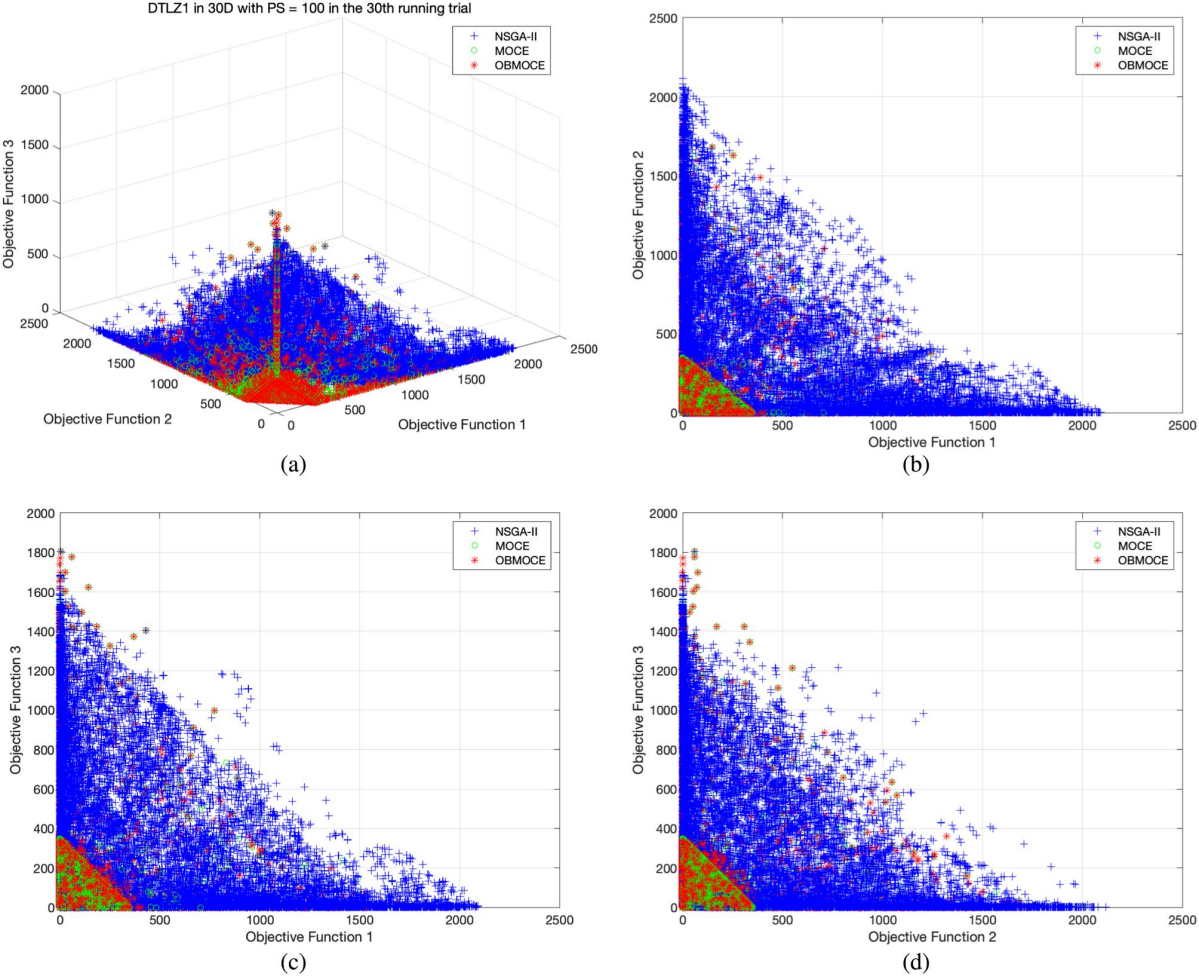
The distribution of solutions derived by three investigated algorithms across all generations from the 30th running trial for DTLZ1 test function, (**a**) plotted in three-dimensional objective space, (**b**) projected plot onto two dimensions as obj1 vs. obj2, (**c**) projected plot onto two dimensions as obj1 vs. obj3, and (**d**) projected plot onto two dimensions as obj2 vs. obj3.

**Figure 5:**
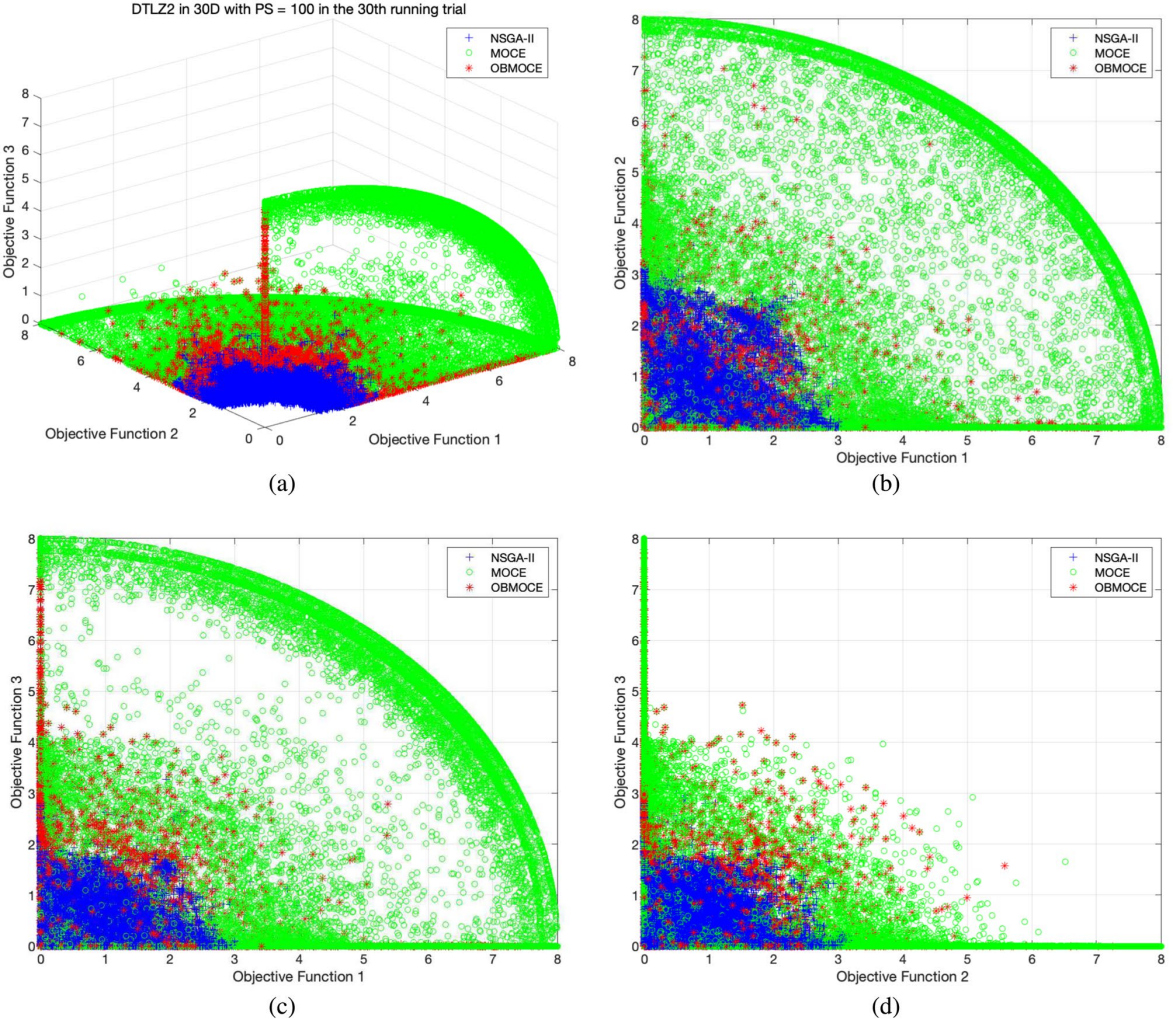
The distribution of solutions derived by three investigated algorithms across all generations from the 30th running trial for DTLZ2 test function, (**a**) plotted in three-dimensional objective space, (**b**) projected plot onto two dimensions as obj1 vs. obj2, (**c**) projected plot onto two dimensions as obj1 vs. obj3, and (**d**) projected plot onto two dimensions as obj2 vs. obj3.

**Figure 6:**
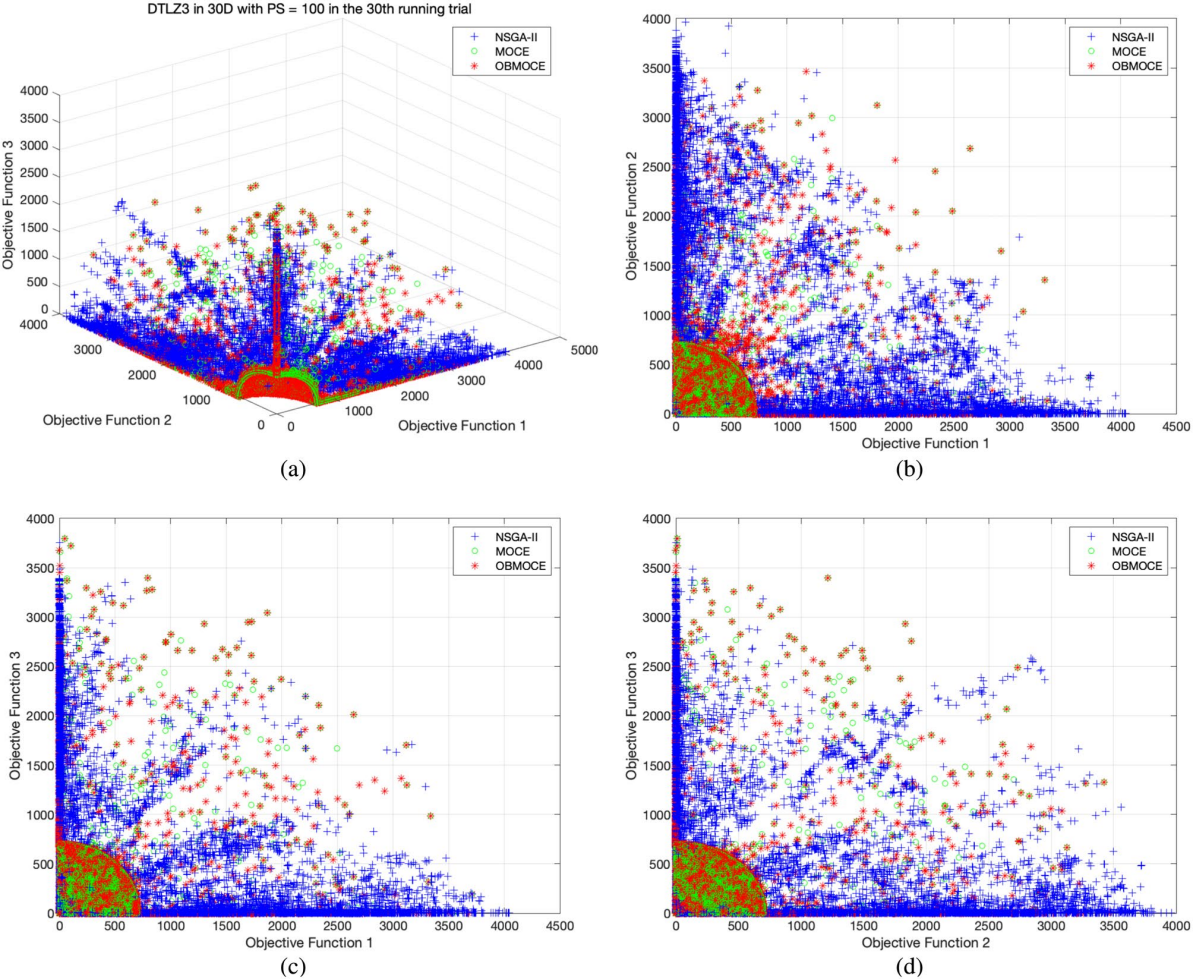
The distribution of solutions derived by three investigated algorithms across all generations from the 30th running trial for DTLZ3 test function, (**a**) plotted in three-dimensional objective space, (**b**) projected plot onto two dimensions as obj1 vs. obj2, (**c**) projected plot onto two dimensions as obj1 vs. obj3, and (**d**) projected plot onto two dimensions as obj2 vs. obj3.

**Figure 7:**
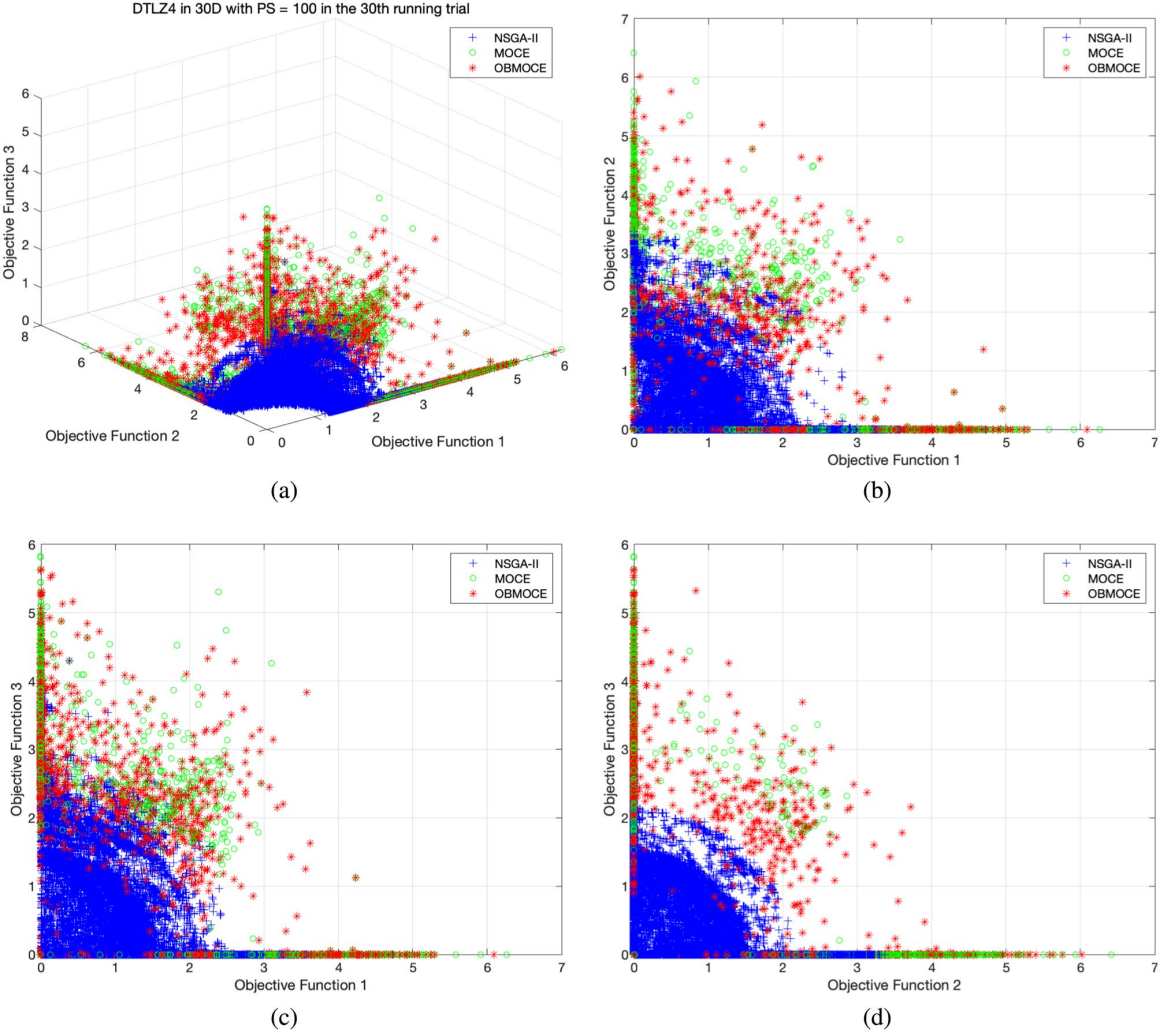
The distribution of solutions derived by three investigated algorithms across all generations from the 30th running trial for DTLZ4 test function, (**a**) plotted in three-dimensional objective space, (**b**) projected plot onto two dimensions as obj1 vs. obj2, (**c**) projected plot onto two dimensions as obj1 vs. obj3, and (**d**) projected plot onto two dimensions as obj2 vs. obj3.

**Figure 8:**
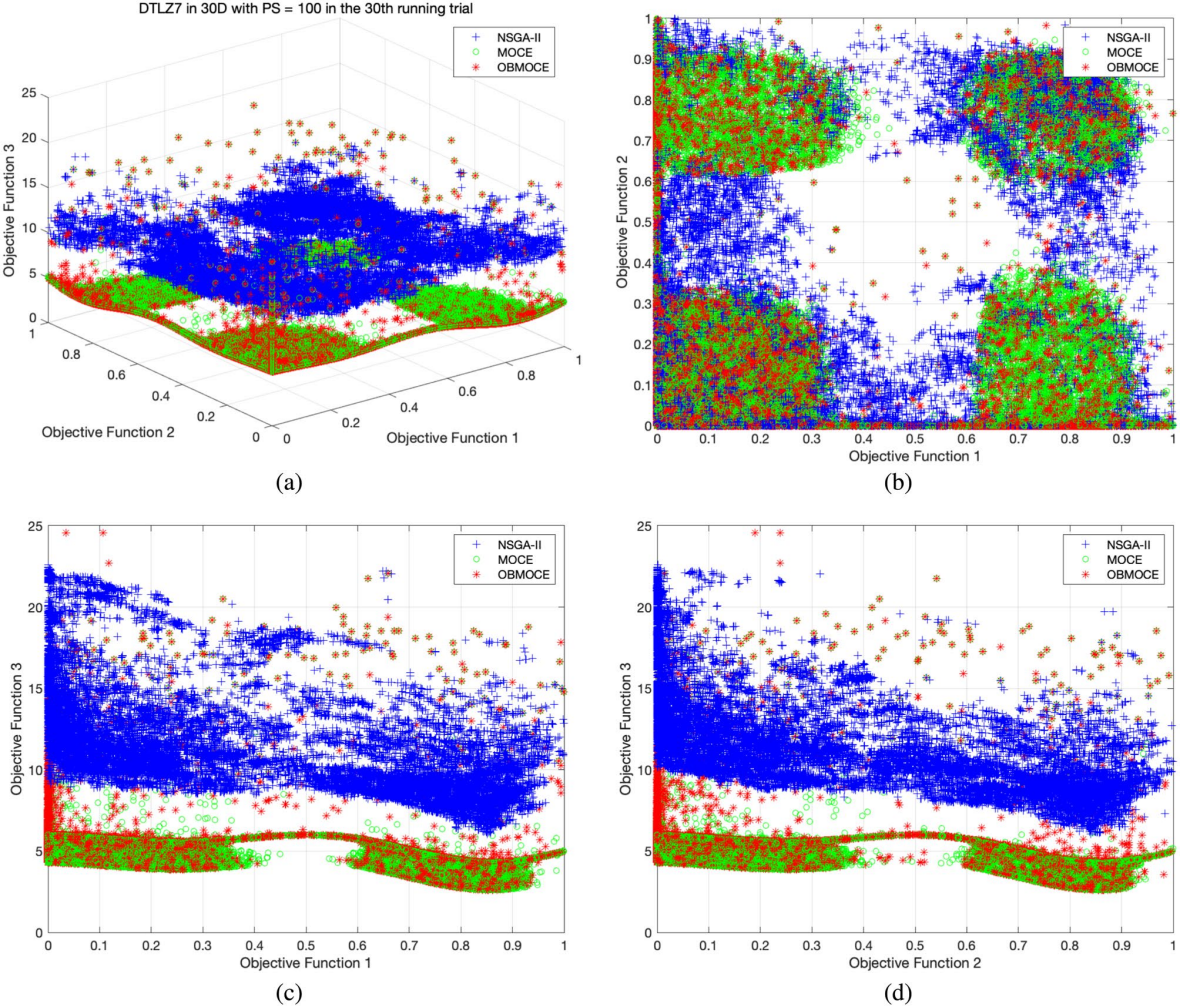
The distribution of solutions derived by three investigated algorithms across all generations from the 30th running trial for DTLZ7 test function, (**a**) plotted in three-dimensional objective space, (**b**) projected plot onto two dimensions as obj1 vs. obj2, (**c**) projected plot onto two dimensions as obj1 vs. obj3, and (**d**) projected plot onto two dimensions as obj2 vs. obj3.

Figures 3, 4, 5, 6, 7, and 8 illustrate the distribution of solutions obtained by the three investigated algorithms across all generations from the 30th trial for ZDT and DTLZ series test functions. Convergence and diversity are two critical performance metrics in the evaluation of multi-objective evolutionary algorithms. Better convergence enables the algorithm to effectively solve practical problems, while improved diversity offers a broader set of trade-off solutions for decision-makers. To compare the proposed OBMOCE algorithm with MOCE and NSGA-II, three widely used evaluation metrics were employed: generational distance (GD)^29^, inverted generational distance (IGD)^30^, and hypervolume (HV)^31^. These metrics were used to assess the convergence and diversity of the Pareto fronts generated by the algorithms.

The GD metric is defined to measure the proximity of the obtained approximation front to the true Pareto front in the objective space. The IGD metric, proposed as an enhancement over GD, evaluates the proximity of the true Pareto front to the obtained approximation front. A smaller distance in either metric indicates that the approximate front is closer to the true Pareto front, implying better convergence performance. The HV metric quantifies the volume of the dominated region in the objective space with respect to a reference point and the approximation front obtained by the algorithm. Larger HV values indicate superior performance, as this metric captures both convergence and diversity characteristics of the approximation fronts.

In this experiment, 50 reference points were selected from the true Pareto front for GD and IGD evaluation. The reference point chosen for HV evaluation was determined based on the upper bound of the union of approximation fronts generated by the three comparison algorithms, ensuring that it is dominated by all obtained solutions.

Since the number of new individuals generated by the proposed OBMOCE algorithm is twice that of the other two algorithms in each generation, it implies that the computational cost of the proposed algorithm OBMOCE is twice that of the conventional MOCE algorithm and NSGA-II algorithm in each generation. Consequently, the number of fitness evaluations is used as the baseline for subsequent performance comparisons.

**Figure 9:**
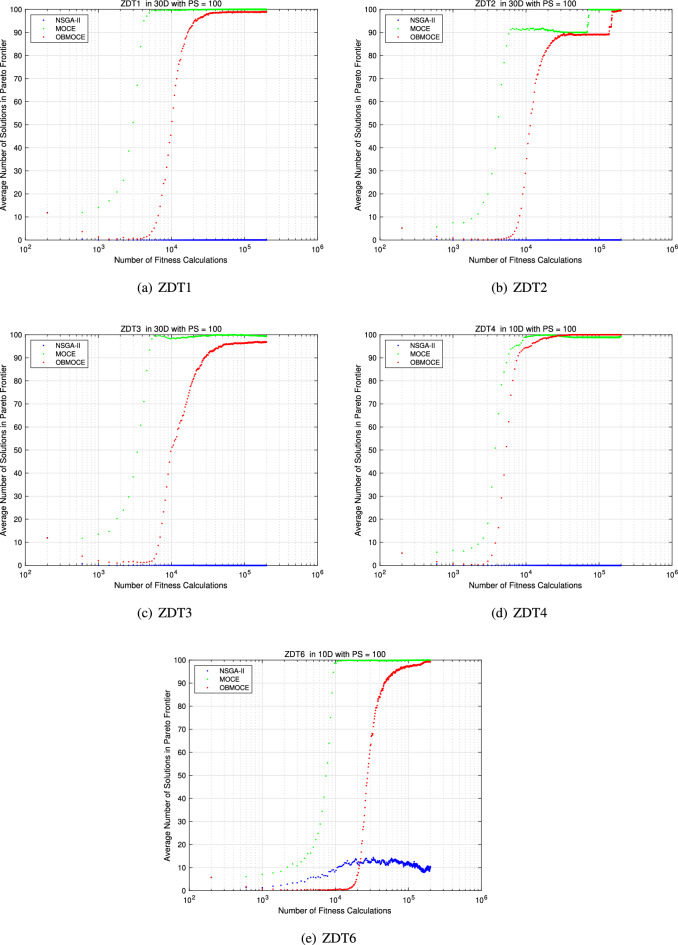
The average number of Pareto frontier solution based on the same computational cost for ZDT series test functions.

**Figure 10:**
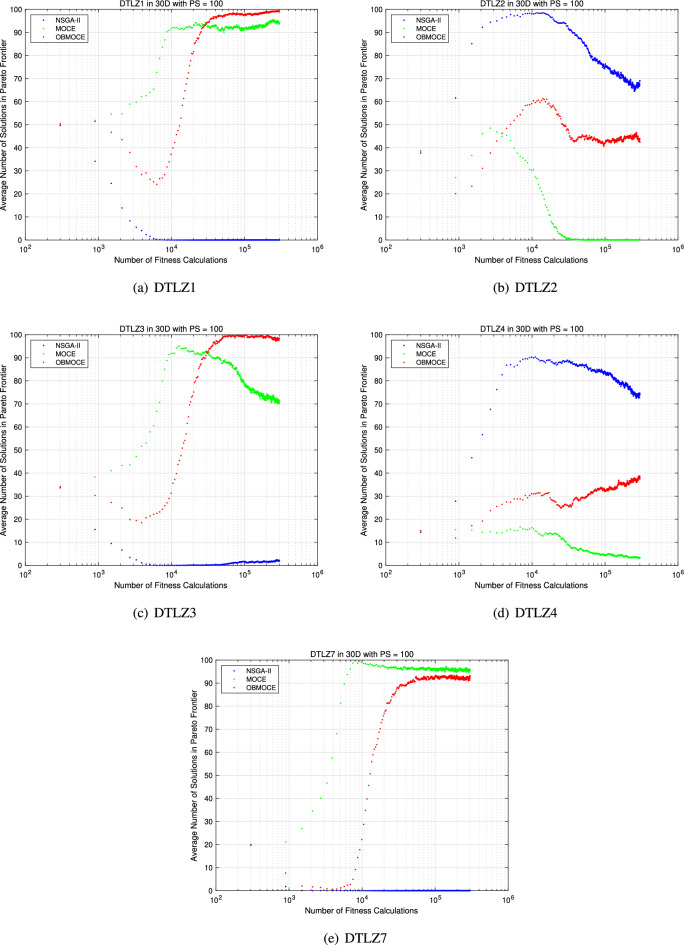
The average number of Pareto frontier solution based on the same computational cost for DTLZ series test functions.

Figures 9 and 10 illustrate the average number of Pareto frontier solutions obtained by the three algorithms, measured under the same number of fitness evaluations. Tables 8, 9, and 10 present the average values of the evaluation metrics GD, IGD, and HV for 10 multi-objective test functions with NSGA-II, MOCE, and OBMOCE algorithms at the 1000th, 1000th, and 500th generations, respectively, across 30 independent trials. As previously noted, the computational cost of evaluating fitness values consumes the most time in the algorithm. These algorithms have the same numbers of fitness calculations in these corresponding generations. To assess the statistical significance of performance differences, the Wilcoxon signed-rank test with a significance level of $\alpha < 0.05$ is employed using two-sided tests.

**Table 8:** Mean GD and Wilcoxon signed-rank test results for 30 running trials.

	NSGA-II	MOCE	OBMOCE
ZDT1	7.8464E−02	8.8000E−04‡	1.7990E−03‡
ZDT2	1.0031E−01	8.2500E−04‡	1.8580E−03‡
ZDT3	4.5882E−02	9.4800E−04‡	9.4500E−04‡
ZDT4	3.3925E+00	8.7000E−04‡	8.8000E−04‡
ZDT6	2.2184E−01	8.3400E−04‡	8.9100E−04‡
DTLZ1	2.2849E+02	5.3359E+01‡£	5.5485E+01‡
DTLZ2	1.0869E−01§£	5.6208E−01	1.2059E−01§
DTLZ3	2.3195E+02	7.0158E+01‡	7.0226E+01‡
DTLZ4	8.4890E−02§£	1.5567E−01	1.2573E−01§
DTLZ7	1.8845E−01	1.0431E−02‡	1.0288E−02‡

$\ddag$, $\S$, and $\pounds$ represent algorithms that are significantly better than NSGA-II, MOCE, and MOCEOB, respectively, according to the Wilcoxon signed-rank test ($p < 0.05$)

**Table 9:** Mean IGD and Wilcoxon signed-rank test results for 30 running trails.

	NSGA-II	MOCE	OBMOCE
ZDT1	4.1873E−01	4.8890E−03‡	4.9350E−03‡
ZDT2	7.8461E−01	7.9197E−02‡	7.9215E−02‡
ZDT3	2.5664E−01	3.3460E−03‡	3.1950E−03‡
ZDT4	2.0167E+01	4.9120E−03‡£	5.3790E−03‡
ZDT6	2.4515E+00	4.4670E−03‡	4.5830E−03‡
DTLZ1	8.8479E+02	4.0642E+02‡£	4.1505E+02‡
DTLZ2	5.1286E−01§£	2.0658E+00	9.2789E−01§
DTLZ3	6.7446E+02	6.9931E+02	6.6658E+02§
DTLZ4	3.5287E−01§£	1.2539E+00	9.9938E−01§
DTLZ7	8.7467E−01	1.2993E−01‡	1.1336E−01‡

$\ddag$, $\S$, and $\pounds$ have the same meanings as in Table 8

**Table 10:** Mean HV and Wilcoxon signed-rank test results for 30 running trials.

	NSGA-II	MOCE	OBMOCE
ZDT1	7.2433E−01	8.9603E−01‡	9.0003E−01‡
ZDT2	5.7700E−01	8.0040E−01‡	8.0790E−01‡
ZDT3	7.7073E−01	9.2843E−01‡	9.2903E−01‡
ZDT4	7.1760E−01	9.9560E−01‡	9.9587E−01‡
ZDT6	1.9473E−01	6.1377E−01‡	6.1470E−01‡
DTLZ1	9.5347E−01	9.9840E−01‡	9.9770E−01‡
DTLZ2	9.9480E−01§£	9.3107E−01	9.8943E−01§
DTLZ3	9.6950E−01	9.9343E−01‡	9.9350E−01‡
DTLZ4	9.0907E−01§£	5.7777E−01	7.1783E−01§
DTLZ7	5.3750E−01	7.5410E−01‡	7.5547E−01‡

$\ddag$, $\S$, and $\pounds$ have the same meanings as in Table 8

Figures 9 and 10 demonstrate that the proposed algorithm OBMOCE significantly outperforms the NSGA-II algorithm in obtaining more Pareto solutions across most multi-objective test functions, particularly evident in the ZDT series test functions. Although OBMOCE initially generates fewer Pareto solutions than the conventional MOCE algorithm, its performance gradually improves with increasing numbers of fitness evaluations and eventually surpasses MOCE in scenarios such as ZDT4, DTLZ1, DTLZ2, DTLZ3, and DTLZ4.

To objectively evaluate the optimization performance of the three algorithms, the Wilcoxon signed-rank test is applied to compare the evaluation metrics (GD, IGD, and HV) at equivalent numbers of fitness evaluations. This non-parametric statistical hypothesis test is used to determine whether significant differences exist between paired algorithmic results.

As shown in Tables 8, 9, and 10, both OBMOCE and MOCE exhibit superior performance compared to NSGA-II across most test functions, with the exception of DTLZ2 and DTLZ4. Moreover, the experimental results suggest that the proposed OBMOCE algorithm significantly outperforms the conventional MOCE algorithm in terms of GD and/or IGD evaluation metrics for the DTLZ2, DTLZ3, and DTLZ4 test functions. Meanwhile, it is worth noting that the proposed OBMOCE algorithm exhibits significantly inferior optimization performance compared to the conventional MOCE algorithm in GD and IGD metrics for the DTLZ1 test function. It demonstrates that integrating the OBL mechanism into a conventional MOCE algorithm can significantly enhance optimization performance in certain cases. However, at times, it may also introduce the side effect of slowing down the optimization speed. Nonetheless, OBMOCE consistently outperforms MOCE in terms of HV across all test functions except DTLZ1. Although the MOCE algorithm exhibits a superior HV value compared to OBMOCE for DTLZ1, this difference is not statistically significant according to the Wilcoxon signed-rank test. Conversely, OBMOCE shows significantly better HV performance than MOCE on DTLZ2 and DTLZ4. These results indicate that integrating the OBL mechanism into the MOCE framework enhances solution diversity.

## OBCE for application in design of hybrid rocket engine

### Experimental setting

To evaluate the effectiveness of the OBL mechanism on the optimization performance of the conventional chaotic evolution algorithm in practical applications, the three multi-objective evolutionary algorithms described in the previous section are applied to the conceptual design optimization problem of a hybrid rocket engine (HRE)^32^. The evaluation module for this optimization task was developed and published by the Japan Aerospace Exploration Agency^33^.

The HRE combines elements of both liquid and solid rocket engines. A key distinction between HRE and traditional rocket engines lies in the differing physical states of the fuel and oxidizer, which result in unique combustion characteristics. This configuration provides several advantages, including improved safety, environmental sustainability, and cost-effectiveness. By leveraging evolutionary computation in numerical simulations and experimental modeling, optimal design parameters for the HRE can be identified efficiently and at relatively low cost.

In this experiment, the evaluation module involves six design variables, as summarized in Table 11. The throttling parameters $a_1$ and $a_2$ are fixed at 0, and the payload mass $M_{pay}$ [kg] is set to 50 kg. Three optimization objectives are considered, as listed in Table 12. Additional details on the fitness evaluation procedures can be found in the original study^33^. The experimental parameters used in this study are provided in Table 13.

**Table 11:** The descriptions of the six design variables used in evaluation module of the HRE conceptual design optimization problem.

Abbreviation	Meaning	Unit	Range
$m_{oxi}$	Initial flow rate of oxidizer	kg/sec	[1.0, 30.0]
$L_{fuel}$	Length of fuel	m	[1.0, 10.0]
$r_{port}$	Initial radius of port	m	[0.01, 0.2]
$t_{burn}$	Combustion time	s	[15.0, 35.0]
$P_{ch}$	Pressure of combustion chamber	bar	[30.0, 40.0]
$\epsilon$	Aperture ratio of nozzle	–	[5.0, 7.0]

**Table 12:** The descriptions of the three studied objectives in the HRE conceptual design optimization problem for this experiment.

Abbreviation	Meaning	Unit	Optimization objective
$M_{tot}$	Initial gross weight	kg	Minimization
$H_{max}$	Highest reachable altitude	km	Maximization
$L_{tot}$	Length of rocket	m	Minimization

**Table 13:** The experimental parameters settings for the three-objective HRE conceptual design optimization problem in this study.

Population size	30
Max. search generation	40, 80
Dimensions of benchmark function	6
Direction factor rate	0.5
Crossover rate	0.9
Num. of trial runs	1

### Results and discussion

**Figure 11:**
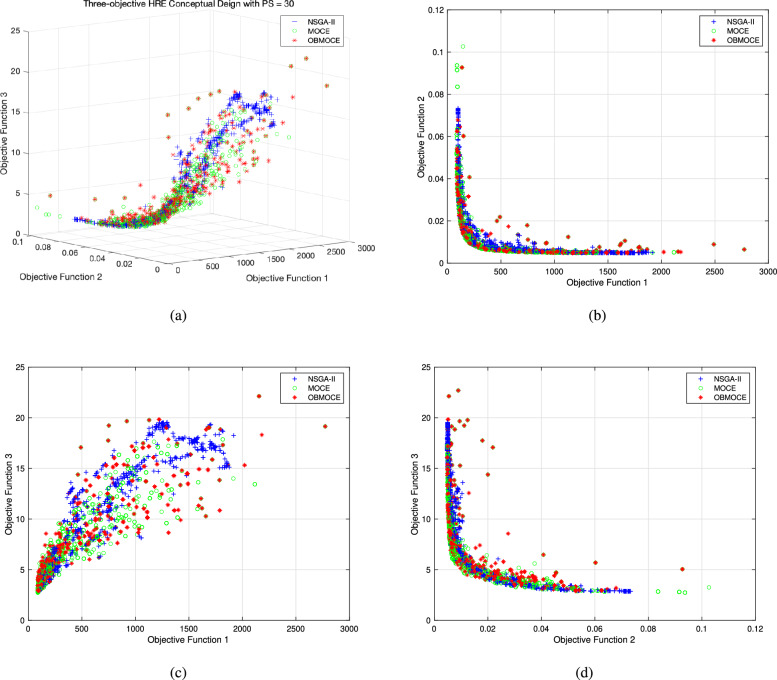
The distribution of solutions derived by three investigated algorithms across all generations for three-objective HRE conceptual design optimization problem, (**a**) plotted in three-dimensional objective space, (**b**) projected plot onto two dimensions as initial gross weight $M_{tot}$(obj1) vs. reciprocal of highest reachable altitude $1/H_{max}$(obj2), (**c**) projected plot onto two dimensions as initial gross weight $M_{tot}$(obj1) vs. length of rocket $L_{tot}$(obj3), and (**d**) projected plot onto two dimensions as reciprocal highest reachable altitude $1/H_{max}$(obj2) vs. length of rocket $L_{tot}$(obj3).

Figure 11 illustrates the distribution of solutions derived by the three investigated algorithms across all generations for the three-objective HRE conceptual design optimization problem. Since the conceptual design optimization problem is still under study, there are no true Pareto front solutions available to evaluate the GD and IGD performance metrics. Hence, the quantity of Pareto frontier solutions and the HV performance metric are utilized to evaluate the three investigated algorithms and analyze the effectiveness of the OBL mechanism on the conventional chaotic evolution algorithm for practical application.

**Figure 12:**
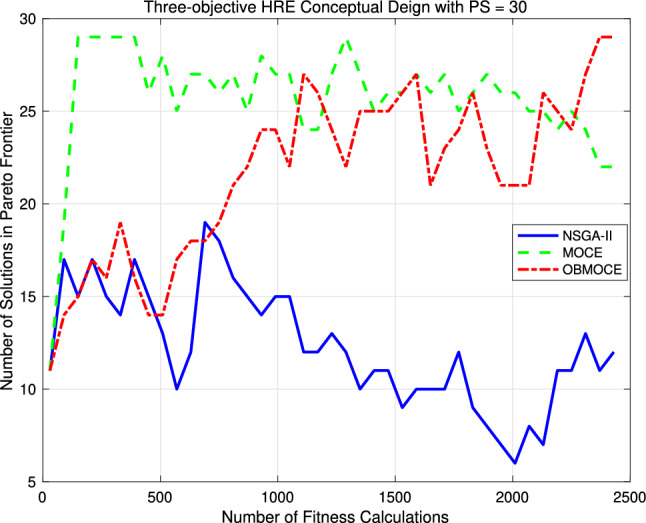
The number of Pareto frontier solution based on the same computational cost for the three-objective HRE conceptual design optimization problem.

Figure 12 presents the number of Pareto frontier solutions obtained using the three investigated algorithms for the HRE conceptual design optimization problem, based on the same fitness calculations. Table 14 shows the values of the HV performance metric for the three-objective HRE conceptual design optimization problem using the NSGA-II, MOCE, and OBMOCE algorithms at the 80th, 80th, and 40th generations, respectively. The corresponding number of fitness calculations remains consistent at 2430 times.

**Table 14:** The HV performance metric for the three-objective HRE conceptual design optimization problem.

	NSGA-II	MOCE	OBMOCE
HRE	7.0400E−01	7.1200E−01	7.2500E−01

As shown in Fig. 12, the performance outcomes of the three algorithms closely resemble those observed in the multi-objective benchmark function optimization presented in the preceding section. Given the same number of fitness evaluations, both the proposed OBMOCE algorithm and the conventional MOCE algorithm yield a greater number of Pareto solutions than the NSGA-II algorithm. While OBMOCE initially generates fewer Pareto solutions than MOCE, its performance improves with increasing numbers of fitness evaluations and eventually surpasses that of MOCE. Table 14 further indicates that the OBMOCE algorithm achieves the highest HV value among the three algorithms, suggesting that it produces a more diverse and convergent set of Pareto-optimal solutions. These experimental findings indicate that incorporating the OBL mechanism into the conventional MOCE algorithm improves the likelihood of generating additional Pareto solutions and enhances solution diversity in practical multi-objective optimization problems.

It is worth emphasizing that while the HRE conceptual design optimization serves as a representative and practically relevant example, the proposed OBCE framework is inherently generalizable. Its diversity-enhancing mechanism and convergence benefits make it suitable for a wide variety of real-world parameter tuning tasks across domains such as mechanical design, machine learning model calibration, and energy system optimization.

## Discussion on mechanism of opposition-based learning

In the proposed OBCE algorithms, the population is iteratively updated to progressively evolve toward the global optimum, aided by the OBL mechanism and a chaotic search strategy. Specifically, the OBL mechanism enhances the search capability of the chaotic evolution algorithm in two primary ways. Firstly, it generates an opposition-based population in each generation to explore the global optimum. Secondly, it furnishes the previously selected opposition-based population for the chaotic search strategy to exploit the global optimum.

To evaluate the effectiveness of the OBL mechanism within the proposed OBCE algorithms, the opposition-based population was monitored, and the best individual within this population was ranked based on fitness values at each generation. Table 15 reports the average rank of the best individual from the opposition-based population across the single-objective benchmark functions. In addition, the best individual was tracked and ranked among the combined set of the current opposition-based population and the offspring generated from the previous opposition-based population, as summarized in Table 16. Lower ranking values indicate superior performance.

As shown in Tables 15 and 16, the opposition-based population demonstrates strong performance in accelerating the optimization process during the early stages, but its competitiveness decreases in the later generations. However, the average ranking of the best individual among the opposition-based population and offspring of the previous opposition-based population consistently maintains a favorable ranking. These findings suggest that the offspring of the opposition-based population consistently plays a significant role in approaching the global optimum. During the initial stages, the OBL mechanism contributes significantly by efficiently directing the search away from premature convergence and unproductive regions toward more promising areas. However, as convergence progresses toward the global or competitive local optima, it becomes apparent that the effectiveness of the OBL mechanism diminishes. While the opposition-based population generated by the OBL mechanism loses competitiveness in the later stages, the offspring of the previous opposition-based population remains potent and competitive in terms of exploitation. This underscores the crucial role that the OBL mechanism objectively plays in facilitating exploration during the early stages.

**Table 15:** Average ranking of the best individual among the opposition-based population in each of the 83 generations over 30 running trials for single-objective benchmark functions.

	F1	F2	F3	F4	F5	F6	F7	F8	F9	F10	F11	F12
(a) 2-dimensional benchmark functions with a population size of 10												
2–84	3.84	4.00	4.51	4.69	3.98	2.61	4.75	1.93	3.77	5.06	2.61	3.69
85–167	4.06	4.32	4.21	4.72	3.95	2.44	4.93	1.92	3.72	5.22	2.56	4.41
168–250	4.10	4.27	4.03	4.88	3.53	2.45	4.94	1.98	3.51	5.25	2.50	4.64
251–333	4.09	4.11	3.75	5.04	3.40	2.54	4.92	2.02	3.52	5.29	2.50	4.69
334–416	4.07	4.22	3.71	5.09	3.35	2.68	4.92	2.03	3.53	5.27	2.50	4.71
417–499	4.08	4.22	3.81	5.21	2.67	2.59	4.92	2.02	3.61	5.18	2.58	4.72
500–582	4.08	4.22	3.92	5.54	2.42	2.59	4.92	2.03	3.61	5.14	2.63	4.77
583–665	4.08	4.22	3.79	5.61	2.41	2.60	4.92	2.05	3.61	5.14	2.63	4.82
(b) 10-dimensional benchmark functions with a population size of 50												
2–84	9.93	7.66	5.26	7.55	7.17	8.80	11.22	1.90	6.35	8.19	2.61	9.36
85–167	12.66	9.35	6.44	10.83	11.00	11.69	14.55	2.13	9.48	9.39	2.76	12.40
168–250	13.20	10.10	6.33	12.41	11.77	13.15	13.66	2.03	10.84	10.15	2.90	13.85
251–333	13.19	11.12	5.96	12.44	10.52	13.39	15.07	1.99	11.50	11.11	2.58	14.19
334–416	13.97	11.68	5.70	13.76	10.67	13.39	16.43	2.05	11.80	11.87	2.81	13.76
417–499	14.44	11.68	6.17	13.80	10.44	15.02	17.13	1.75	12.17	12.48	2.86	14.26
500–582	14.45	11.46	6.09	14.32	10.77	15.80	17.27	1.72	12.14	12.61	2.71	15.19
583–665	14.55	11.17	6.49	12.97	10.80	15.91	17.01	1.69	12.54	12.53	3.04	15.90
(c) 30-dimensional benchmark functions with a population size of 150												
2–84	21.89	9.46	9.13	8.22	10.84	26.15	17.08	2.55	10.22	15.66	2.41	18.89
85-167	34.62	10.79	11.00	9.07	13.62	40.18	24.11	2.11	14.46	22.68	2.82	27.70
168–250	44.86	11.62	13.11	10.84	15.19	45.87	27.39	2.16	15.43	25.48	3.02	25.96
251–333	48.90	11.18	13.65	11.59	17.87	51.67	28.77	2.22	16.60	26.78	2.84	29.15
334–416	51.45	11.01	14.02	11.24	18.40	53.86	32.12	2.28	17.41	27.89	2.96	30.12
417–499	52.26	11.35	13.52	11.08	20.51	52.24	32.52	2.20	17.90	29.18	3.00	31.17
500–582	53.25	12.23	11.16	13.35	22.14	54.24	31.24	1.94	19.49	29.85	3.18	28.78
583–665	54.29	12.09	10.64	14.51	22.22	56.45	32.29	2.07	19.72	31.22	2.75	28.33

**Table 16:** Average ranking of the best individual among the opposition-based population and offspring of previous opposition-based population in each of the 83 generations over 30 running trials for single-objective benchmark functions.

	F1	F2	F3	F4	F5	F6	F7	F8	F9	F10	F11	F12
(a) 2-dimensional benchmark functions with a population size of 10												
2–84	1.68	1.82	1.63	1.98	1.53	1.52	2.12	1.26	2.14	2.01	1.40	1.62
85–167	1.68	1.72	1.46	1.85	1.32	1.20	2.20	1.20	1.91	1.91	1.26	1.60
168–250	1.60	1.74	1.47	1.75	1.26	1.22	2.21	1.19	1.80	1.93	1.21	1.61
251–333	1.60	1.70	1.42	1.66	1.19	1.24	2.21	1.15	1.80	1.94	1.20	1.61
334–416	1.60	1.72	1.39	1.66	1.16	1.27	2.21	1.13	1.80	1.98	1.20	1.60
417–499	1.62	1.72	1.40	1.64	1.10	1.27	2.21	1.09	1.83	2.00	1.20	1.60
500–582	1.63	1.72	1.39	1.63	1.10	1.27	2.21	1.07	1.83	2.02	1.20	1.60
583–665	1.63	1.72	1.29	1.63	1.07	1.27	2.21	1.07	1.83	2.04	1.20	1.60
(b) 10-dimensional benchmark functions with a population size of 50												
2–84	1.40	1.64	1.67	1.61	1.84	2.10	1.76	1.26	1.87	1.86	1.44	1.65
85–167	1.25	1.34	1.44	1.30	1.69	1.95	1.48	1.18	1.63	1.76	1.11	1.30
168–250	1.24	1.33	1.34	1.22	1.63	1.86	1.40	1.14	1.64	1.78	1.09	1.33
251–333	1.21	1.35	1.33	1.14	1.60	1.86	1.40	1.13	1.57	1.65	1.07	1.31
334–416	1.20	1.37	1.29	1.17	1.57	1.83	1.40	1.15	1.56	1.59	1.05	1.32
417–499	1.22	1.39	1.19	1.18	1.55	1.80	1.40	1.10	1.57	1.60	1.07	1.34
500–582	1.23	1.40	1.13	1.17	1.56	1.82	1.40	1.10	1.57	1.60	1.07	1.32
583–665	1.23	1.39	1.13	1.13	1.49	1.83	1.40	1.10	1.57	1.61	1.07	1.34
(c) 30-dimensional benchmark functions with a population size of 150												
2–84	1.84	1.80	1.59	1.56	1.48	2.00	1.79	1.35	1.59	1.99	1.31	1.99
85–167	1.58	1.68	1.38	1.42	1.36	2.00	1.49	1.13	1.32	1.68	1.16	1.67
168–250	1.38	1.61	1.36	1.37	1.40	1.71	1.47	1.08	1.41	1.73	1.12	1.67
251–333	1.44	1.55	1.39	1.38	1.43	1.63	1.52	1.06	1.47	1.57	1.10	1.70
334–416	1.35	1.42	1.39	1.34	1.41	1.68	1.55	1.03	1.47	1.43	1.11	1.60
417–499	1.27	1.30	1.43	1.32	1.46	1.60	1.55	1.03	1.39	1.42	1.11	1.62
500–582	1.27	1.14	1.47	1.31	1.52	1.57	1.48	1.03	1.40	1.42	1.10	1.62
583–665	1.32	1.15	1.43	1.33	1.47	1.60	1.37	1.03	1.40	1.44	1.10	1.60

Similar experiments were conducted on multi-objective benchmark functions and the three-objective HRE conceptual design optimization problem using non-dominated sorting and crowding distance. The results of these experiments are presented in Tables 17, 18, 19, and 20. Based on the experimental findings, it can be concluded that the OBL mechanism provides a favorable trade-off between computational cost and performance during the early stages of the optimization process. However, as the search progresses, the mechanism tends to incur higher computational costs with diminishing returns. These observations suggest that incorporating more effective strategies for detecting premature convergence within the population may improve the efficiency of OBL usage. Accordingly, determining when and how to activate the OBL mechanism in a more adaptive and targeted manner will be prioritized in future work.

**Table 17:** Average ranking of the best individual among the opposition-based population in each of the 50 generations over 30 running trials for multi-objective benchmark functions with a population size of 100. - represents that no individual selected from the opposition-based population during these corresponding generations.

	ZDT1	ZDT2	ZDT3	ZDT4	ZDT6	DTLZ1	DTLZ2	DTLZ3	DTLZ4	DTLZ7
1–50	60.62	49.92	55.07	2.18	51.83	2.07	2.27	2.14	2.27	50.74
51–100	–	–	–	2.04	–	1.72	2.05	1.25	2.56	–
101–150	–	–	–	2.11	–	1.62	2.08	1.19	2.73	–
151–200	–	–	–	2.10	–	1.52	1.97	1.28	2.85	–
201–250	–	–	–	2.10	–	1.47	2.12	1.49	2.94	–
251–300	–	2.00	–	2.11	–	1.51	2.24	1.58	2.95	–
301–350	–	2.00	–	2.10	–	1.61	2.29	1.58	2.75	–
351–400	3.00	2.00	–	2.10	–	1.64	2.19	1.56	2.69	–
401–450	2.00	2.10	–	2.11	–	1.72	2.23	1.57	2.62	–
451–500	2.50	2.14	–	2.10	–	1.75	2.20	1.60	2.93	–

**Table 18:** Average ranking of the best individual among the opposition-based population and offspring of previous opposition-based population in each of the 50 generations over 30 running trials for multi-objective benchmark functions with a population size of 100.

	ZDT1	ZDT2	ZDT3	ZDT4	ZDT6	DTLZ1	DTLZ2	DTLZ3	DTLZ4	DTLZ7
1–50	6.44	6.46	6.52	1.40	12.41	1.16	1.30	1.20	1.24	7.02
51–100	2.81	2.18	4.50	1.06	4.87	1.06	1.00	1.00	1.00	1.93
101–150	3.05	1.62	2.06	1.00	1.25	1.05	1.00	1.00	1.00	1.79
151–200	2.03	1.45	1.45	1.00	1.25	1.03	1.00	1.00	1.00	1.69
201–250	1.73	1.36	1.49	1.00	1.25	1.05	1.00	1.00	1.00	1.63
251–300	1.59	1.30	1.15	1.00	1.07	1.08	1.00	1.00	1.00	1.58
301–350	1.23	1.33	1.13	1.00	1.00	1.07	1.00	1.00	1.00	1.61
351–400	1.57	1.44	1.13	1.00	1.00	1.07	1.00	1.00	1.00	1.61
401–450	1.69	1.73	1.13	1.00	1.00	1.06	1.00	1.00	1.00	1.67
451–500	1.73	2.14	1.14	1.00	1.00	1.03	1.00	1.00	1.00	1.75

**Table 19:** Average ranking of the best individual among the opposition-based population in each of the 4 generations for HRE conceptual design optimization problem with a population size of 30. - represents that no individual selected from the opposition-based population during these corresponding generations.

	HRE conceptual optimization problem
1–4	5.50
5–8	5.33
9–12	–
13–16	30.00
17–20	–
21–24	–
25–28	–
29–32	–
33–36	–
37–40	–

**Table 20:** Average ranking of the best individual among the opposition-based population and offspring of the previous opposition-based population in each of the 4 generations for HRE conceptual design optimization problem with a population size of 30.

	HRE conceptual optimization problem
1–4	5.50
5–8	2.75
9–12	2.00
13–16	2.00
17–20	1.25
21–24	4.75
25–28	8.50
29–32	9.00
33–36	4.25
37–40	3.50

The primary contribution of the OBL mechanism lies in increasing the likelihood of exploring potential search areas and alleviating premature convergence. In the conventional chaotic evolution algorithm, the approach towards the global or local optimum involves randomly searching for potentially superior solutions within a specified range of the target variable in each generation. However, when an individual becomes trapped in a local optimum, and its search range is insufficient to escape the region dominated by that optimum, it becomes unlikely for the individual to deviate from its current position. As a result, the algorithm continues to perform redundant and ineffective computations until the final generation. Conversely, expanding the search range may increase the risk of overlapping with those of other target variables. In such overlapping regions, certain variables are more likely to be selected, although their fitness values may not be superior to those in unexplored regions. This can again lead to redundant and unnecessary calculations. By integrating the OBL mechanism into the conventional chaotic evolution algorithm, these limitations can be effectively mitigated. Specifically, the OBL mechanism allows for the strategic use of convergence information to discover new candidate solutions, which helps to escape premature convergence, accelerate optimization, and improve solution diversity.

## Conclusion and future work

This study introduces the OBL mechanism to the conventional chaotic evolution algorithm and evaluates its effectiveness across diverse optimization scenarios, including single-objective benchmark function optimization, multi-objective benchmark function optimization, and a practical multi-objective application optimization. Experimental results demonstrate that the OBL mechanism significantly improves the convergence speed and optimization performance of the conventional chaotic evolution algorithm for single-objective tasks. Additionally, integrating the OBL mechanism into the conventional MOCE algorithm for multi-objective tasks increases the likelihood of improving both the quantity and diversity of solutions on the Pareto frontier.

The results presented in this study confirm that OBCE is a versatile algorithm with strong potential for broader applicability. While the evaluation in this paper focuses on benchmark problems and a hybrid rocket engine design task, the OBCE framework can be readily extended to other real-world applications that require the optimization of complex, high-dimensional, and multi-modal objective landscapes. In particular, OBCE is well suited for scenarios involving parameter tuning, making it applicable to a wide range of engineering and decision-making problems.

The primary contribution of this work lies in incorporating a lightweight scheme, known as OBL, to enhance the conventional chaotic evolution algorithm. The proposed OBCE algorithms demonstrate that the OBL mechanism plays a vital role in reducing unnecessary computational cost while improving solution quality in both single-objective and multi-objective tasks.

Future research will focus on developing advanced strategies and mathematical mechanisms to further address premature convergence and enhance the performance of evolutionary algorithms. Promising directions include leveraging convergence and density information from existing Pareto solutions to identify more competitive search regions, optimizing parameter configurations, and refining the integration of surrogate-assisted and diversity-guided techniques.

## Data Availability

No datasets were generated or analysed during the current study.
